# Case Report: Lethal neonatal hypertrophic cardiomyopathy from compound heterozygous *MYBPC3* variants

**DOI:** 10.3389/fcvm.2025.1726463

**Published:** 2025-12-18

**Authors:** Jianying Wang, Lingye Hong, Yao Li, Zhengrong Mao, Yuefeng Zhu, Ming Qi, Ren Zhou, Xutao Hong

**Affiliations:** 1 Forensic Science Center of Zhejiang University and Zhejiang University School of Medicine, Hangzhou, China; 2 Department of Preventive Medicine, School of Public Health, Wenzhou Medical University, Wenzhou, China; 3 Affiliated Sir Run Run Shaw Hospital, Zhejiang University School of Medicine, Hangzhou, China; 4 Yikon Genomics Co., Ltd, Shanghai, China

**Keywords:** atrial septal defect, case report, genotype-phenotype correlation, hypertrophic cardiomyopathy, molecular autopsy, MYBPC3, systems medicine

## Abstract

**Introduction:**

Bi-allelic pathogenic variants in *MYBPC3* cause a rare and lethal neonatal form of hypertrophic cardiomyopathy (HCM) that often evades detection during routine prenatal screening. We report a comprehensive investigation of such a case to highlight the clinical utility of postmortem molecular diagnosis.

**Methods:**

A two-month-old infant died from sudden-onset acute heart failure. We performed a full forensic autopsy with detailed histological examination and conducted trio-based whole-exome sequencing (WES) on the proband and parents to identify the genetic etiology.

**Results:**

Postmortem examination revealed severe HCM, an atrial septal defect (ASD), and extensive myocardial necrosis and fibrosis. WES identified compound heterozygous pathogenic variants in *MYBPC3*: a known paternal splice-site variant (c.2905+1G>A) and a novel maternal truncating frameshift variant (c.836del; p.Gly279Valfs*21). Both variants are predicted to result in a complete loss of protein function.

**Discussion:**

This “molecular autopsy” established a definitive cause for the infant's death, linking a novel variant to a severe pathological phenotype. Crucially, the diagnosis guided the clinical management of the asymptomatic carrier parents, prompting long-term cardiac surveillance and enabling preimplantation genetic testing (PGT) for future family planning. This case demonstrates how integrating molecular diagnostics with forensic pathology facilitates a systems medicine approach, transforming a fatal index case into actionable preventive care for the entire family.

## 1 Introduction

Hypertrophic cardiomyopathy (HCM) is the most prevalent inherited cardiac muscle disorder, primarily caused by pathogenic variants in genes encoding sarcomeric proteins. Among these, variants in the *MYBPC3* gene, which encodes cardiac myosin-binding protein C (cMyBP-C), are the most frequent cause. The classic presentation of *MYBPC3*-related HCM is an autosomal dominant disease characterized by incomplete penetrance. Most heterozygous carriers typically develop symptoms in **adulthood**, often after the third decade of life, presenting with dyspnea, chest pain, arrhythmias, and an increased risk of heart failure and sudden cardiac death (1, 2).

While historically considered a disease of adults, it is now recognized that sarcomeric gene variants are also the leading cause of non-syndromic HCM in children, with up to two-thirds of pediatric cases having an identifiable sarcomeric mutation (3). Childhood-onset HCM, though less common, is associated with considerable life-long morbidity and a higher risk of sudden cardiac death compared to adult-onset disease (3). An even rarer and more severe inheritance pattern involves bi-allelic pathogenic variants, where an individual inherits a deleterious variant from each parent. This homozygous or compound heterozygous state leads to a dramatically more severe clinical course. Specifically, bi-allelic truncating mutations in *MYBPC3* have been shown to cause a severe, often lethal, neonatal cardiomyopathy, characterized by very early onset, aggressive biventricular hypertrophy, and frequently accompanied by other structural cardiac anomalies, such as septal defects (4).

These severe neonatal cases pose a significant diagnostic challenge, as routine prenatal screening may fail to detect evolving cardiac abnormalities. Here, we report a fatal case of a neonate with HCM and an atrial septal defect (ASD) resulting from compound heterozygous *MYBPC3* variants. This case adds to the growing body of evidence on the devastating impact of bi-allelic *MYBPC3* mutations by providing detailed pathological insights linked to a novel pathogenic variant and critically highlights the need for integrating advanced diagnostic modalities into prenatal and postnatal care.

## 2 Case presentation

### 2.1 Patient information and clinical findings

The patient was a male infant born at 40 weeks of gestation to a healthy, non-consanguineous couple (mother aged 25, father aged 31) with an unremarkable family history. The birth weight was 3.6 kg. All routine prenatal ultrasound examinations during pregnancy were reported as normal. Age at diagnosis was 2 months, when the infant presented with a sudden onset of lethargy, pallor, and cyanosis. Upon arrival at the emergency department, the infant was in critical condition with severe respiratory distress and circulatory collapse. Due to the rapid clinical deterioration and immediate need for resuscitation, a detailed physical examination, including cardiac auscultation, could not be fully documented. However, signs of acute heart failure, including central cyanosis and poor perfusion, were noted prior to cardiac arrest. Despite immediate resuscitation efforts, he succumbed to acute heart failure within two hours of presentation. A timeline of the clinical course, diagnostic assessment, and follow-up is presented in Table 1.

**Table 1:** Timeline of case history and management.

Time point	Event/intervention	Outcome/findings
Prenatal period	Routine obstetric ultrasounds (Trimesters 1–3)	No cardiac abnormalities detected; normal fetal growth and anatomy reported.
Birth (day 0)	Spontaneous vaginal delivery at 40 weeks	Male infant; birth weight 3.6 kg; Apgar scores normal; discharged as healthy.
Postnatal day 57	Vaccination	Administered oral pentavalent rotavirus vaccine.
Postnatal day 58	Symptom onset	Developed lethargy, decreased milk intake, and increased defecation frequency.
Postnatal day 60	Acute deterioration & death	Symptoms worsened rapidly; admitted with acute heart failure; resuscitation failed; death occurred within 2 h.
Postmortem (day 61)	Forensic and Pathological Autopsy	Gross finding: cardiomegaly. Samples fixed in formalin due to pending legal/consent process.
2 Months post-death	Consent for Genetic Testing	Parents agreed to genetic testing after resolution of initial disputes.
3 Months post-death	Genetic Analysis Report	Trio-WES identified compound heterozygous*MYBPC3*variants.
3.5 Months post-death	Genetic Counseling & Family Screening	Parents recalled; confirmed as carriers; initiated cardiac surveillance (mild mitral regurgitation noted).
11 Months post-death	Reproductive Planning	Couple initiated IVF cycle with Preimplantation Genetic Testing (PGT).

### 2.2 Pathological and genetic findings

**Figure 1:**
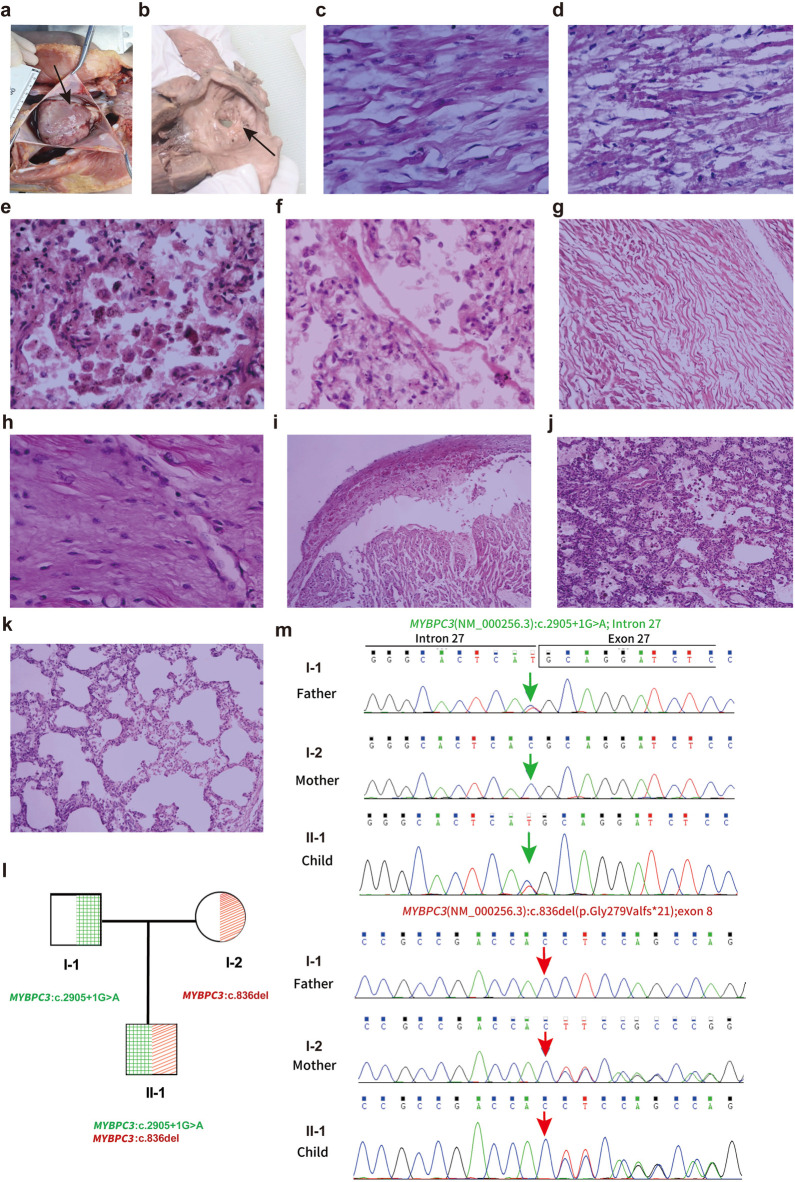
Autopsy findings and histological analysis of the cardiac abnormalities. **(a)** Heart morphology demonstrating overall enlargement, particularly of the right atrium and right ventricle (HP:0001667). **(b)** Observation of a defect at the foramen ovale measuring 0.7 cm × 0.3 cm, noted alongside a defect in the left atrial side of the foramen ovale membrane. **(c)** Histological examination showing myocardial hypertrophy (HE 40X). **(d)** Myocardial contraction band necrosis (HE 10X). **(e)** Alveoli containing phagocytic cells and heart failure cells indicative of pulmonary congestion (HE 40X). **(f)** Hyaline membrane formation in the lung (HE 40X). **(g)** Myocardial twisting (HE 10×), **(h)** Myocardial fibrosis (HE 40×), **(i)** Myocardial hemorrhage with myocardial vacuolar degeneration (HE 10×), **(j)** Pulmonary hemorrhage (HE 10×), **(k)** Pulmonary emphysema (HE 10×). **(l,m)** Sanger sequencing validation of *MYBPC3*variants in the proband and parents. with one variant inherited from the father and the other from the mother. Exon boundaries and numbers are indicated above the chromatograms. Note that sequences are shown from the reverse strand.

A comprehensive postmortem evaluation was performed with parental consent. Gross pathological examination revealed significant cardiomegaly with marked right ventricular hypertrophy and an atrial septal defect (Figures 1a,b). Histological analysis confirmed extensive myocardial hypertrophy with disorganized cardiomyocytes, contraction band necrosis, and significant interstitial fibrosis (Figures 1c,d,h). The final pathological diagnosis was congenital heart disease (HCM and ASD) leading to death from acute heart failure (Table 2).

**Table 2:** Clinical data.

Case	Parental details			Age at diagnosis	Phenotypes (HPO terms)^a^	Obstetric history	Family history	Outcome
1	Maternal	Age	25	Two months	hypertrophic cardiomyopathy (HP:0001639), atrial septal defect (HP:0001631), acute heart failure (HP:0001635)	G1P1	Unremarkable	Age at diagnosis: 2 months, the neonate was found to have hypertrophic cardiomyopathy (HP:0001639) and an atrial septal defect (HP:0001631), ultimately succumbing to acute heart failure (HP:0001635). Died aged 2 month
		Ethnicity	Asian					
	Paternal	Age	31					
		Ethnicity	Asian					

a HPO (Human Phenotype Ontology) provides a standardized vocabulary of phenotypic abnormalities to facilitate precise clinical description.

Given the non-specific presentation of sudden neonatal death and the broad differential diagnosis encompassing cardiomyopathies, channelopathies, and metabolic disorders, trio-based whole-exome sequencing (WES) was selected over a targeted gene panel. This approach provided an unbiased genomic evaluation to maximize diagnostic yield and allowed for the immediate confirmation of variant inheritance and segregation, which is critical in identifying compound heterozygous etiologies in acute settings. Variant interpretation followed the American College of Medical Genetics and Genomics (ACMG) guidelines (5). WES identified two pathogenic variants in the *MYBPC3* gene: a paternal splice-site variant (NM_000256.3: exon 27; c.2905+1G>A) and a novel maternal frameshift deletion (NM_000256.3:exon 8; c.836del; p.Gly279Valfs*21). The novel c.836del variant is absent in large population databases (e.g., gnomAD), supporting its pathogenicity (PM2_P criterion). Sanger sequencing validated the compound heterozygous state in the proband and the carrier status of the parents (Figures 1l,m; Table 3).

**Table 3:** Genetic findings

Procedure (Age at diagnosis)	Performed test	Secondary confirmatory test	Gene (name; REFSEQ)	Known disease (OMIM)	Variant	ACMG classify-cation	Criteria applied^a^	Inheritance & zygosity	Interpretation
Two months	WES	Sanger sequencing	*MYBPC3*: NM_000256.3	hypertrophic cardiomyopathy (omim:115197)	c.2905+1G>A	Pathogenic	PVS1+PS4+PM2_p	AD: heterozygous	Consistent with symptoms
Two months	WES	Sanger sequencing	*MYBPC3*: NM_000256.3	hypertrophic cardiomyopathy (omim:115197)	c.836del (p.Gly279Valfs*21)	Likely pathogenic	PVS1+PM2_p	AD: heterozygous	Consistent with symptoms

a PVS1 (Pathogenic Very Strong): Null variant (nonsense, frameshift, canonical ±1 or 2 splice sites, initiation codon, single or multiexon deletion) in *MYBPC3* gene where loss of function (LOF) is a known mechanism of disease. PS4 (Pathogenic Strong): The prevalence of the variant in affected individuals is significantly increased compared with the prevalence in controls. PM2_P (Pathogenic Moderate - Supporting): Absent from controls (or at extremely low frequency) in population databases (e.g., gnomAD), used here as a supporting line of evidence.

## 3 Discussion

This case report describes a severe, neonatal-lethal presentation of HCM and ASD driven by compound heterozygous pathogenic variants in the *MYBPC3* gene. This bi-allelic inheritance pattern deviates from the classic autosomal dominant model associated with adult-onset HCM and explains the extreme severity and early onset of the disease.

Mechanistically, both identified variants are predicted to result in a complete loss of function. The paternal splice-site variant (c.2905+1G>A) disrupts a canonical donor site, leading to exon skipping or intron retention. As LOF is a known mechanism of disease for *MYBPC3*, the PVS1 criterion is applicable, supporting its classification as ‘Pathogenic’. The novel maternal frameshift variant (c.836del; p.Gly279Valfs*21) occurs in exon 8 (of 35), introducing a premature termination codon (PTC) in the early coding sequence. Transcripts containing such PTCs are typically targeted for degradation by nonsense-mediated decay (NMD), preventing the production of truncated protein. Consequently, this compound heterozygous state likely results in a “null” phenotype with a near-total absence of functional cMyBP-C protein. While we aimed to confirm protein absence via Western blot, protein extraction from the autopsy tissue was unsuccessful due to prolonged formalin fixation necessitated by legal proceedings prior to consent. This highlights a practical challenge in forensic cases and underscores the value of early fresh-frozen tissue banking for functional validation.

Our histopathological findings provide a structural correlate to this molecular diagnosis and offer a distinct comparison to previously reported cases. This case provides a more detailed pathological insight that strengthens the genotype-phenotype correlation. While Wessels *et al*. described myofibrillar disarray and hypertrophy in a neonate with biallelic truncating *MYBPC3* mutations (Patient 1), they specifically noted an absence of significant interstitial fibrosis (4). In contrast, our case revealed not only extensive myocardial disarray—a direct consequence of cMyBP-C deficiency—but also significant interstitial fibrosis and contraction band necrosis (Figures 1d,h). The presence of necrosis (indicative of acute ischemic injury) alongside fibrosis (indicative of chronic remodeling) in a two-month-old infant suggests an exceptionally aggressive pathophysiology. The complete lack of cMyBP-C likely rendered the sarcomeres incapable of handling normal hemodynamic stress, leading to rapid myocyte energy depletion, acute injury, and accelerated maladaptive remodeling.

A critical aspect of this case is the failure of routine prenatal ultrasound to detect any cardiac abnormalities. This highlights a limitation of standard prenatal screening. While some studies demonstrate the utility of high-resolution ultrasonography (6–8), the nuanced diagnosis of fetal HCM often requires a more specialized approach. Expert fetal echocardiography can identify early signs of cardiomyopathy, such as ventricular hypertrophy, septal defects, or evidence of diastolic dysfunction, sometimes as early as the second trimester (9). Advanced techniques, including speckle-tracking echocardiography, have further enhanced diagnostic capabilities by detecting subtle impairments in myocardial function even before overt hypertrophy becomes visible. The prenatal identification of HCM allows for crucial parental counseling, delivery planning at a tertiary care center with neonatology and cardiology support, and facilitates immediate postnatal management (10). However, a key limitation is that HCM can be a progressive condition *in utero*. A normal fetal echocardiogram in mid-gestation does not entirely exclude the possibility of the disease manifesting later in the third trimester or the neonatal period, as was likely the situation in our case. This suggests that for high-risk pregnancies, such as those with a known familial pathogenic variant, serial fetal echocardiograms may be warranted.

Beyond clarifying the proband's etiology, the most significant contribution of this investigation lies in its application of a systems medicine framework to familial risk management. The genetic diagnosis in the proband had profound and positive implications for the parents. As both parents were identified as heterozygous carriers of a pathogenic *MYBPC3* variant, they were alerted to their own risk for developing adult-onset HCM and potential sudden cardiac death. Although still young, subsequent cardiac evaluations revealed minor mitral valve regurgitation in both, but no significant hypertrophy as of yet. This early warning has prompted them to enroll in a long-term cardiac monitoring program, which is vital for the timely prevention and management of HCM. This demonstrates the immense value of a “reverse-phenotyping” approach, where a child's diagnosis directly informs the health management of the parents.

Furthermore, armed with this definitive genetic information, the couple has made informed reproductive choices. They have initiated an *in vitro* fertilization (IVF) cycle at a reproductive center, intending to utilize preimplantation genetic testing (PGT) to select an unaffected embryo for transfer. This proactive measure will prevent the recurrence of this devastating condition in future offspring, transforming reproductive outcomes for families with known monogenic risks.

## 4 Conclusion

We report a fatal case of neonatal-onset hypertrophic cardiomyopathy caused by compound heterozygous *MYBPC3* variants. This case powerfully underscores the critical importance of performing a comprehensive postmortem evaluation, including both traditional autopsy and molecular genetic testing (a “molecular autopsy”), in all cases of unexplained neonatal death. The integration of these approaches not only clarified the precise genetic etiology of the severe neonatal phenotype but also delivered crucial, actionable insights for the family. The diagnosis has enabled proactive cardiac surveillance for the asymptomatic carrier parents and, critically, provided an accurate recurrence risk, empowering them to pursue PGT to ensure the health of future children. This highlights that a thorough investigation following a pediatric tragedy can offer a clear roadmap for long-term health and reproductive planning for the entire family.

## Data Availability

Due to patient privacy and ethical restrictions regarding the pediatric subject and family members, the raw genetic data cannot be deposited in a public repository. The data supporting the findings are available from the corresponding author upon reasonable request.
